# A Comprehensive Characterisation of Mycelium-Based Biomaterials Developed from *Panus ciliatus* and *P. subfasciatus* (Panaceae, Polyporales)

**DOI:** 10.3390/jof11120826

**Published:** 2025-11-22

**Authors:** Sabin Khyaju, Kevin D. Hyde, Kitiphong Khongphinitbunjong, Sitthi Duangphet, Worawoot Aiduang, Thatsanee Luangharn

**Affiliations:** 1 Center of Excellence in Fungal Research, Mae Fah Luang University, Chiang Rai 57100, Thailand; 6571105505@lamduan.mfu.ac.th (S.K.);; 2 School of Science, Mae Fah Luang University, Chiang Rai 57100, Thailand; 3 Mushroom Research Foundation, Bandu, Chiang Rai 57100, Thailand; 4 Center of Innovative Materials for Sustainability, Mae Fah Luang University, Chiang Rai 57100, Thailand; 5 Office of Research Administration, Chiang Mai University, Chiang Mai 50200, Thailand; 6 Department of Biology, Faculty of Science, Chiang Mai University, Chiang Mai 50200, Thailand

**Keywords:** biobased material, mycelium-based application, new geographical record, sustainable materials

## Abstract

Mushroom mycelium-based biomaterials (MMBs) are sustainable materials derived from fungal species and lignocellulosic substrates. In this study, fresh specimens of *Panus ciliatus* and *P. subfasciatus* collected from mixed deciduous forests in Chiang Rai Province, Thailand, were identified through a combination of morphological characteristics and phylogenetic analyses based on the ITS region. *Panus ciliatus* is reported as a new geographical record from Thailand. This is the first comprehensive study on MMB samples developed using mycelia of these species and rubber sawdust for their physical, mechanical, hydrodynamic, and chemical properties. Additional analyses included FTIR spectroscopy, thermogravimetric analysis, flammability testing, and soil burial degradability. Based on the material properties and successfully developed prototypes, the MMBs are potential in packaging, indoor uses, construction, and insulation purposes, as an alternative to conventional synthetic materials. Notably, Ashby chart of mechanical properties showed the MMB could substitute foam. Thermogravimetric analysis of MMB showed thermal stability with weight loss approximately 50–60% at 293–298 °C. Soil burial of MMB for 90 days shows cumulative weight loss exceeding 60% proving biodegradable. Additionally, a new approach for mycelial viability maintenance is described and verified, addressing the problem to maintain vigorous mycelium.

## 1. Introduction

*Panus* Fr. (Panaceae, Polyporales) was described by Fries [1], with *P. conchatus* (Bull.) Fr. as the type species. Members of this genus are wood-inhabiting saprobes distributed worldwide, particularly in subtropical and tropical regions [2,3]. *Panus* species are characterised by a centrally to eccentrically stipitate basidiomata; infundibuliform to cyathiform pilei; a hymenophore with decurrent lamellae, free gills; ellipsoid, smooth, thin-walled, inamyloid basidiospores; thin to thick-walled cystidia; and a dimitic hyphal system with unbranched skeletal hyphae [1,3,4].

*Panus* was treated as an infrageneric taxon of *Lentinus* Fr., and classified as *Lentinus* subg. *Panus* (Fr.) Pegler [5,6]. Subsequent molecular phylogenetic analyses supported its recognition as a distinct genus within the family Panaceae [7]. Panaceae comprises only two genera, namely *Cymatoderma* Jungh. and *Panus* [2,8]. Index Fungorum has listed 255 records of *Panus* (https://www.indexfungorum.org/names/Names.asp, accessed on 22 October 2025). However, only 20 species of *Panus* are recognised worldwide [9,10]. Some species, including *P. conchatus*, *P. neostrigosus* Drechsler-Santos & Wartchow, *P. rudis* Fr., and *P. strigellus* (Berk.) Chardón & Toro, are considered edible [11,12,13].

Mushrooms are valued globally for their nutritional, medicinal, and functional properties, with growing production and economic importance [14]. In recent years, mushroom mycelium has also been explored in material science, particularly in the development of MMBs. These materials are eco-friendly, sustainable, and biodegradable, which align with the principles of the circular economy or closed-loop system. Petroleum-based materials, such as plastics and foams, are environmental pollutants that are non-sustainable, non-biodegradable, and leave a significant carbon footprint [15,16]. These materials pose threats to human health, as well as to marine and wildlife. Conversely, MMBs are cost-effective, energy-efficient, non-toxic, and non-polluting materials used as an alternative to plastic- and foam-based products [17,18]. Interestingly, these materials have been produced commercially to be applied in packaging, construction, insulation, architectural design, and leather [16]. However, only a limited number of wood-decaying fungal species, belonging to the genera *Ganoderma*, *Pleurotus*, and *Trametes*, have been investigated and characterised for MMBs development [19].

Among several orders of Basidiomycota, members of the order Polyporales are highly prioritised due to their inherent ability to produce superior-quality MMBs [20,21]. *Panus*, like many other mushroom genera, remains underexplored in the context of MMB development. Interestingly, this genus meets the recommended fungal criteria for developing MMBs as reported by Sydor et al. [22], including a saprobic nature, white-rot decay, rapid hyphal growth, a dimitic hyphal system, and non-toxicity. Additionally, Hibbett et al. [23] demonstrated that *Panus* mycelia grow effectively on sawdust at room temperature and a moisture content of 65%. In this study, wild strains of *Panus ciliatus* and *P. subfasciatus* collected from forests of Chiang Rai Province, Thailand, were identified through a combination of morphological and molecular phylogenetic analyses (ITS region). Subsequently, *Panus ciliatus* is reported as a new geographical record from Thailand. The pure mycelium from these species was used to develop MMBs, which were subsequently evaluated for their physical (moisture content, density, shrinkage), mechanical (compression, flexural, tensile, and impact strengths), hydrodynamic (water absorption, water contact angle), and chemical (pH, electrical conductivity, organic matter, organic carbon, nitrogen, phosphorus, and potassium contents) properties. This is the first report of MMBs produced from these fungi. Additional evaluations included Fourier-transform infrared (FTIR) spectroscopy, thermogravimetric analysis (TGA), flammability, soil burial degradability, and maintenance of mycelial viability. Additionally, MMB prototypic products were successfully developed, demonstrating their potential in the valorization of lignocellulosic wastes and applications in packaging, construction, insulation, and indoor uses.

## 2. Materials and Methods

### 2.1. Specimen Collection and Isolation

Fresh specimens of *Panus ciliatus* and *P. subfasciatus* were collected from decaying logs in a mixed deciduous forest in Chiang Rai Province, Thailand. Macro-morphological characters were recorded from the fresh specimens, which were then air-dried at 55 °C for 48 h until completely dehydrated [21]. The dried specimens were kept in paper bags, stored at room temperature, and subsequently used for micro-morphological characterisation. Fresh specimens were also used for tissue isolation on potato dextrose agar (PDA) plates, which were incubated at 25 °C in the dark. After 3–5 days of growth, cultures were sub-cultured onto a fresh PDA plate to obtain pure isolates. These pure cultures were maintained at 4 °C for further studies.

### 2.2. Macro- and Micro-Morphological Descriptions

Macro-morphological characters were examined both in the field and the laboratory. Fresh specimens were photographed in the field using a Nikon (Tokyo, Japan) SLR D3400 camera. Morphological descriptions followed the methods of Lodge et al. [24], and colour codes and terms were noted according to the Methuen Handbook of Colour [25]. Micro-morphological characters (basidia, basidiospores, cystidia, pileipellis, and hyphae) were observed using OLYMPUS (Tokyo, Japan) BX-53 and Nikon (Tokyo, Japan) Eclipse Ni compound microscopes. Freehand sections of dried specimens were treated with 5% KOH and then mounted in 1% Congo red. Additional sections were mounted in distilled water and/or 5% KOH to determine the presence of pigments or any colour reactions. Basidium length was measured excluding sterigmata. Thirty basidiospores were measured in side view to determine size ranges. The size and shape of basidiospores were expressed by the following Q = L/W ratio: L = mean length, W = mean width. Photomicrographs were taken with a camera attached to the compound microscope, and all microscopic measurements were taken using the Tarosoft^®^ (Bangkok, Thailand) Image Framework program v.0.9.7. Dried specimens were deposited in the Fungarium of Mae Fah Luang University (MFLU), and the pure cultures were deposited in the Mae Fah Luang University Culture Collection (MFLUCC).

### 2.3. DNA Extraction, PCR Amplification, and DNA Sequencing

Genomic DNA was extracted from pure cultures using the High Pure PCR Template Preparation Kit (Roche) following the manufacturer’s protocol. DNA quality and concentration were assessed using a NanoDrop One Microvolume UV-Vis spectrophotometer (Thermo Scientific, Waltham, MA, USA). The ITS region was amplified using the primer pair ITS1/ITS4 [26]. Polymerase chain reaction (PCR) reactions were carried out in a total volume of 25 µL, containing 12.5 µL of 2× Power Taq PCR Master Mix, 1 µL of each primer (20 µM), 1 µL of genomic DNA, and 9.5 µL of deionized water. Amplifications were performed on an Eppendorf Mastercycler (Eppendorf AG 22331 Hamburg, Germany) with the following cycling program: initial denaturation at 94 °C for 3 min, followed by 35 cycles of 95 °C for 30 s, 55 °C for 1 min, and 72 °C for 1 min; and a final extension at 72 °C for 10 min. PCR products were sequenced by SolGent Co., Ltd., Yuseong-gu, Daejeon, Republic of Korea.

### 2.4. Sequence Alignments and Phylogenetic Analyses

Sequence quality was assessed using BioEdit v.7.0.9 [27], and sequences were assembled using Geneious 9.1.2 [28]. The final consensus sequences were deposited in GenBank (https://www.ncbi.nlm.nih.gov/genbank/, accessed on 27 March 2025) to obtain accession numbers for the ITS region. To determine the closest matches, each sequence was compared against the National Center for Biotechnology Information (NCBI) database using online version of the Basic Local Alignment Search Tool (BLAST). The ITS dataset includes newly generated sequences, closely related taxa, available sequences of other species within the genus, and outgroup sequences of *Cymatoderma elegans* (Dai 17511, CBS 491.76), which are used for phylogenetic analysis and reconstruction (Table 1).

Sequence alignment was performed with MAFFT v7 [29] using G-INS-I iterative refinement methods. Ambiguously aligned regions were removed using trimAl v1.4.1 [30], and Gblocks v0.91b [31] was used to refine alignment further, allowing smaller final blocks, gaps position within blocks, and less strict flanking positions. Single-gene phylogenetic analyses were performed for species delimitation. Maximum Likelihood (ML) analyses were conducted in RAxML-GUI v2.0 under the GTRGAMMA substitution model [32]. ML analysis was conducted using the rapid bootstrap algorithm with 1000 replicates. ML bootstrap percentages (MLB) of 70% or greater were considered significant. Gaps were treated as missing data. Bayesian Inference (BI) analyses were conducted using MrBayes v3.2.6 [33] with four Markov chain Monte Carlo (MCMC) chains run for 1,000,000 generations, sampling every 100th generation. The first 25% of trees were discarded as burn-in. Chain convergence was assessed using Tracer v1.5 [34], ensuring effective sample size (ESS) values > 200. Posterior probability (BPP) values ≥ 0.90 indicate strong support. Phylogenetic trees were visualised in FigTree v1.4.4 (http://tree.bio.ed.ac.uk/software/figtree/, accessed on 16 November 2025) and edited in Adobe Photoshop v8.0 (Adobe Systems, USA). The fungal species description followed the guidelines of He et al. [35].

**Table 1:** Fungal name, voucher, geographic origin, and GenBank accession numbers of taxa used in the phylogenetic analysis.

Fungal Species	Voucher	Geographic Origin/Country	GenBank Accession No. (ITS)	References
*Panus baishanzuensis*	FJAU67793.1	China	PP273986	[36]
*P. baishanzuensis*	FJAU67793	China	PP273985	[36]
*P. bambusinus* Type	AK61b	India	MW453097	[2]
*P.* cf. *tephroleucus*	VOG36	Brazil	MT669124	[3]
***P. ciliatus***	**MFLUCC 25-0173**	**Thailand**	**PV393836**	**This study**
*P. ciliatus*	SP446150	Brazil	MT669118	[36]
*P. conchatus*	KUMCC18 0047	China	MK192053	[37]
*P. conchatus*	CLZhao 1452	China	MG231758	[3]
*P. conchatus*	Inat36307978	USA	OM349507	[3]
*P. conchatus*	LE265028	Russia	KM411463	[38]
*P. conchatus*	CBS 267.58	Germany	MH857778	[3]
*P. conchatus*	UOC-MINNP-M13	Sri Lanka	KP776992	[3]
*P. conchatus*	JZ30	India	MG719287	[3]
*P. conchatus*	A52	Philippines	OM102535	[3]
*P. conchatus*	UOC:SIGWI:S24	Sri Lanka	KR818817	[3]
*P. fulvus*	DS1687	Brazil	MT669122	[36]
*P. lecomtei*	HHB 9614	USA	KP135329	[39]
*P. lecomtei*	HHB-11042-Sp	USA	KP135328	[39]
*P. minisporus*	FJAU67792	China	PP273980	[36]
*P. minisporus*	FJAU67792.1	China	PP273981	[36]
*P. neostrigosus*	LSPQ NSM 106	Canada	KU761234	[40]
*P. neostrigosus*	LSPQ NSM 107	Canada	KU761235	[40]
*P. parvus*	URM80840	Brazil	MT669125	[36]
*P. purpuratus*	PDD 96130	New Zealand	MK404671	[2]
*P. roseus*	HKAS 94714	China	KY490136	[2]
*P. rudis*	ZJ1005DKJ02	China	KU863049	[37]
*P. rudis*	ZJ1005DKJ03	China	KU863050	[37]
*P. similis*	LE287548	Vietnam	KM411466	[38]
*P. similis*	FJAU67794	China	PP273984	[36]
*P. sribuabanensis*	SDBR-CMUNK0930	Thailand	OR447475	[2]
*P. sribuabanensis*	SDBR-CMUNK0924	Thailand	OR447474	[2]
*P. strigellus*	B6	Paraguay	MW407012	[2]
*P. subfasciatus* Type	MFLU 16 2129	Thailand	LT614958	[41]
*P. tephroleucus*	CMINPA 1860	Brazil	MN602052	[3]
*P. velutinus*	CM10	Brazil	MT669138	[3]
*P. velutinus*	VOG30	Brazil	MT669139	[36]
*P. strigellus*	INPA222827	Brazil	JQ955722	[3]
***P. subfasciatus***	**MFLUCC 25-0172**	**Thailand**	**PV393835**	**This study**
*Panus* sp.	TAC2130a MO506033	USA	OR336204	GenBank
*Panus* sp.	G028	China	KJ195662	[3]
*Cymatoderma elegans* (outgroup)	Dai 17511	China	ON417155	[2]
*Cymatoderma elegans* (outgroup)	CBS 491.76	Japan	JN649340	[2]

Note: The newly generated sequences are indicated in bold.

### 2.5. Evaluation of Culture Media for Optimal Mycelial Growth

Five agar-based media were tested: corn meal agar (CMA), malt extract agar (MEA), oatmeal agar (OMA), potato dextrose agar (PDA), and yeast malt agar (YMA). All equipment and media were sterilised at 15 psi for 20 min at 121 °C. Mycelial plugs (5 mm diameter) were taken from the actively growing edge of 10-day-old PDA cultures and inoculated onto the test media. Plates were incubated at 25 °C in the dark for 12 days. The mycelium growth rate was recorded daily using the method described by Luangharn et al. [42]. The mycelium density was scored as very scanty (1+), scanty (2+), moderate (3+), somewhat abundant (4+), or abundant (5+) [42]. The growth rate and density measurements were performed in five replicates.

### 2.6. Evaluation of Mycelial Growth in Lignocellulose Substrates

Rubber sawdust (≤2 mm particle size) was soaked in tap water overnight and subsequently drained to remove excess moisture. The sawdust was used as the primary substrate (80% *w*/*w*), supplemented with 10% rice bran, 4% yeast extract, 4% molasses, 1.5% calcium carbonate (CaCO_3_), and 0.5% gypsum (CaSO_4_ · 2H_2_O). A 30 g portion of the prepared substrate mixture was transferred into glass plates, packed in a polypropylene bag, and sterilised at 121 °C for 60 min. After cooling, 5 mm mycelial plugs from 10-day-old cultures of *Panus ciliatus* and *P. subfasciatus* were inoculated at the centre of each plate, and incubated in the dark at 25 °C. Each treatment was performed in five replicates. Mycelial growth diameter was measured every two days, beginning on day 2 post-inoculation, until complete colonisation of the substrate was observed. Mycelial density was recorded on day 14.

### 2.7. Preparation of Test Samples

Test samples were developed from selected fungal species through a series of steps, including spawn preparation, substrate bag formulation, mycelium-based biomaterial fabrication, and denaturation, as described below.

#### 2.7.1. Preparation of Spawn

Spawn preparation followed the method described by Aiduang et al. [19] with modifications. Sorghum grains (*Sorghum bicolor*) were thoroughly washed and soaked in tap water overnight, then boiled for 15 min. After cooling and air-drying at room temperature, 150 g of grains were transferred into sterile glass bottles and autoclaved at 121 °C for 30 min. Once cooled, mycelial plugs of the mushroom strains were aseptically inoculated into the bottles. The inoculated bottles were incubated in the dark at 28 °C for 25 days or until the grains were fully colonised by mycelium.

#### 2.7.2. Preparation of Mycelium Substrate Bags

Bag preparation followed the method described by Luangharn et al. [43] with modifications. The rubber sawdust was used as the primary substrate (80% *w*/*w*), supplemented with 10% rice bran, 4% yeast extract, 4% molasses, 1.5% calcium carbonate, and 0.5% gypsum. The ingredients were manually mixed to achieve a moisture content of 55–60%. A 700 g portion of the mixture was packed into polypropylene bags, sealed with a plastic ring and lids, and sterilised in an autoclave at 121 °C for 60 min. After cooling to room temperature, 20 g of prepared spawn was aseptically inoculated into each bag. The inoculated bags were incubated in the dark at 25 ± 1 °C until complete colonisation.

#### 2.7.3. Preparation of Mould

For the compression strength test, moulds were designed from acrylic transparent sheets that were cut into a cubic shape (5 × 5 × 5 cm), following the method by Özdemir et al. [44]. The moulds for the tensile and impact tests were designed into a dumbbell-shaped segment (165 × 19 mm, neck 57 × 13 mm) and a cuboid shape (63.5 × 12.7 × 12.7 mm), respectively, following American Society for Testing and Materials (ASTM) standards [45]. For the flexural strength test, the moulds were prepared into a cuboid shape (12 × 5 × 5 cm). Glass Petri-dishes (diam. 9 cm) were used to prepare circular samples for FTIR spectroscopy.

#### 2.7.4. Fabrication of Mycelium-Based Biomaterials

Once the sawdust substrate in the bag was fully colonised, the mycelial-substrate mass was broken apart, transferred, and manually pressed into a sterile acrylic mould to form test samples. The moulded mycelium-substrate mixture was incubated for 4–7 days to grow within the mould, then unmoulded and incubated for an additional 4–7 days to allow mycelial growth on surfaces previously in contact with the mould. All the equipment was thoroughly washed in tap water and sterilized by heat treatment in a hot air oven at 60 °C overnight. The procedures were conducted under sterile conditions in a laminar hood to prevent contamination.

#### 2.7.5. Denaturation

**Figure 1:**
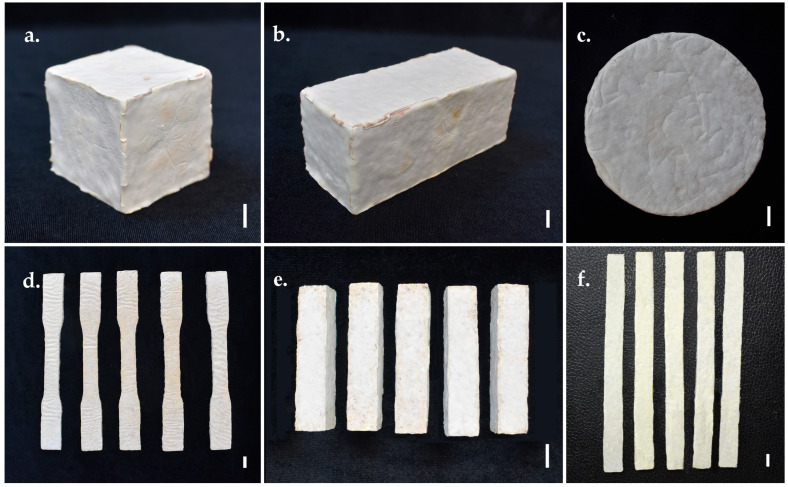
Test samples of MMBs were developed from *Panus ciliatus* (MFLUCC 25-0173) and sawdust: (**a**) Sample prepared for testing moisture content, dry density, volumetric shrinkage, compression strength, and water absorption. (**b**) Sample for flexural strength, SEM, TGA, chemical analyses (pH, organic matter, organic carbon, NPK), and soil burial degradability. (**c**) Sample for FTIR spectroscopy. (**d**) Sample for tensile strength and water contact angle. (**e**) Sample for impact strength. (**f**) Sample for flammability. Scale bars = 1 cm.

The fully colonised mycelium-based composite samples were deactivated by drying in a hot-air oven at 60 °C for 2 days [46]. After cooling to room temperature, the samples were subjected to analyses of physical, mechanical, hydrodynamic, and chemical properties (Figure 1).

### 2.8. Evaluation of Mycelium-Based Biomaterial Test Samples

#### 2.8.1. Physical Properties

##### Moisture Content and Dry Density

The dry density of the MMBs was determined in accordance with the International Organization for Standardization (ISO) 9427:2003. Density was calculated as the ratio of oven-dry mass to volume. Test samples were prepared in the form of cubes with dimensions of 5 × 5 × 5 cm. The test was conducted in five replicates.

##### Volumetric Shrinkage

Fully colonised MMB cubes (5 × 5 × 5 cm) were used to calculate volumetric shrinkage following the method described by Elsacker et al. [47]. Shrinkage percentage was calculated as: shrinkage (%) = (V_1_ − V_2_)/V_1_ × 100, where V_1_ = moist volume of sample and V_2_ = dry volume of sample. The test was conducted in five replicates.

#### 2.8.2. Mechanical Properties

##### Compression Strength

**Figure 2:**
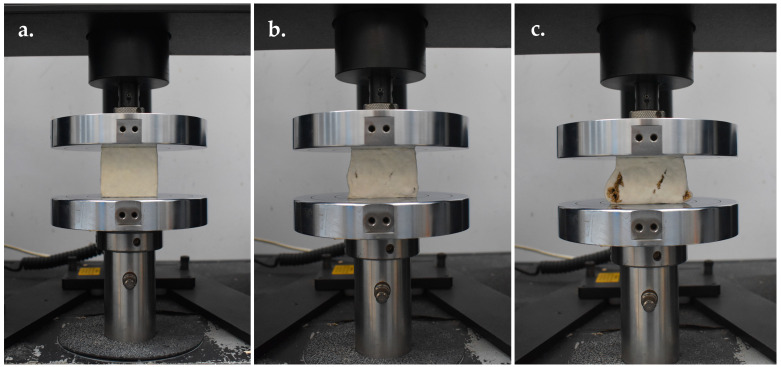
Compressive strength test of MMB developed from *Panus ciliatus* (MFLUCC 25-0173) and sawdust. (**a**) Sample before testing. (**b**) Bulging during compression. (**c**) Cracking.

Compression strength was measured according to the American Society for Testing and Materials (ASTM) D3501-94, following the procedure described by Elsacker et al. [47], using a Universal Testing Machine (UTM; Instron Model 5566, Instron Corporation, Norwood, MA, USA) (Figure 2). The test was conducted with a 10 kN capacity load bench and a 10 kN load cell, operated under ambient conditions at 25 °C, with ⁓50% relative humidity. Tests were performed under displacement control at a rate of 5 mm min^−1^, with five replicates per sample. Compression strength was expressed in kilopascals (kPa) and calculated from the load–displacement data using the following formulas: compressive stress (*σ*) = *F*/*A* and strain (ɛ) = (∆*L*/*L*_o_), where *F* = compressive force (N), *A* = original cross-sectional area (m^2^), Δ*L* = displacement of the loading surfaces (m), and *L*_o_ = original height of the specimen (m).

##### Flexural Strength

**Figure 3:**
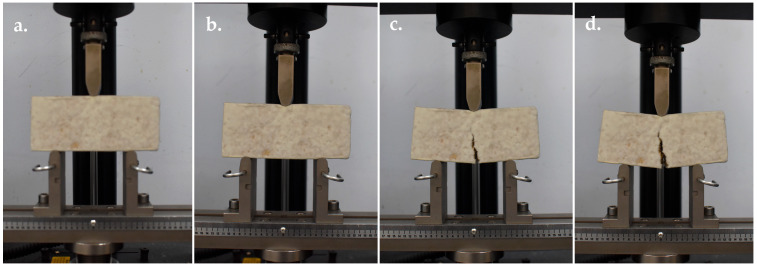
Flexural strength test of MMB developed from *Panus ciliatus* (MFLUCC 25-0173) and sawdust. (**a**) Sample before testing. (**b**) Bending under load. (**c**) Initial crack formation. (**d**) Complete crack.

Flexural strength was conducted according to ASTM D790-10, following the procedure described by Appels et al. [48], using a Universal Testing Machine (UTM; Instron and Model 5566) (Figure 3). The test was performed with a crosshead speed of 2 mm min^−1^ and a clamp support span of 100 mm. Each strain was tested in five replications. Flexural strength was expressed in kilopascals (kPa) and calculated from the load–displacement data using the formulas: flexural stress (*σf*) = 3*FL*/(2*bd*^2^), where *F* = applied load at fracture (N), *L* = support span (m), *b* = specimen width (m), and *d* = specimen thickness (m).

##### Tensile Strength

**Figure 4:**
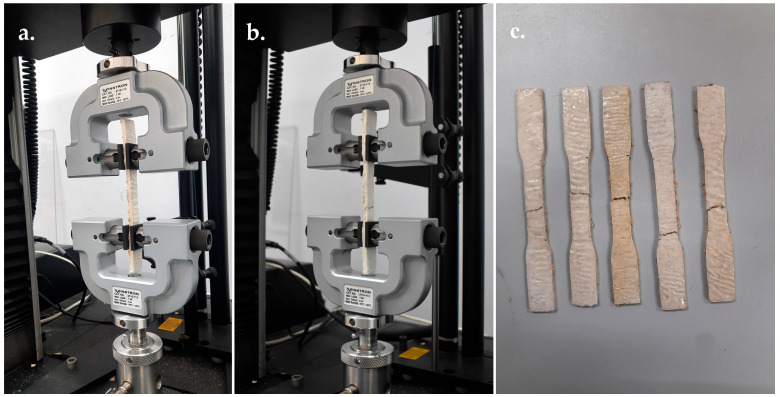
Tensile strength test of MMB developed from *Panus ciliatus* (MFLUCC 25-0173) and sawdust. (**a**) Application of tensile force on the sample. (**b**) Beginning of a crack on the sample. (**c**) Sample after crack.

Tensile strength was measured according to ASTM D638-14, following the procedure described by Appels et al. [48], using a Universal Testing Machine (UTM; Instron Model 5566). Tests were operated at a crosshead speed of 2 mm min^−1^ and a maximum load capacity of 1 kN, under ambient conditions at 25 °C, with ⁓50% relative humidity (Figure 4). Samples were held in the grips of the tensile testing machine and pulled until fracture occurred. Tensile stress was recorded in relation to strain and plotted as a stress–strain curve. Each strain was tested in five replicates. Tensile strength was expressed in kilopascals (kPa) and calculated from the load–displacement data using the formulas: tensile stress (*σt*) = *F*/*A*, where *F* = maximum tensile force at fracture (N) and *A* = original cross-sectional area of the specimen (m^2^).

##### Impact Strength

Impact strength was measured using the Charpy impact test according to ASTM D256, following the procedure described by Aiduang et al. [45], using an Impact Pendulum Machine (Instron; Model CEAST 9050). Samples were loaded in the testing machine and subjected to a pendulum strike until fracture occurred. Each strain was tested with five replicates. Impact strength (IS) was calculated as: *IS* = *K*/*A*, where *K* = energy required to fracture the sample (kJ) and *A* = cross-sectional area of the sample (m^2^).

#### 2.8.3. Hydrodynamic Properties

##### Water Absorption

Water absorption rate was measured following ASTM C272/C272M-18, as described by Attias et al. [49], by partially submerging the MMB samples in water at room temperature (25 °C, 50% RH). The water absorption rate was determined based on the change in weight relative to the initial dry weight.

##### Water Contact Angle

The water contact angle (CA) was measured using a contact angle meter (Kino SL200KS, Puditec, Bangkok, Thailand) following the procedure described by Antinori et al. [50]. A 5 μL droplet of deionised water was statically deposited on the sample surfaces of each sample, and side-view images of the droplets were captured at 10 and 60 s. Each treatment was replicated 15 times.

#### 2.8.4. Chemical Properties

The MMB samples were ground into a powder form using a blender and sieved to obtain particles smaller than 2 mm. The pH of the samples was determined following Alemu et al. [51] by mixing 5 g of each sample in 50 mL Milli-Q water for 1 h and measuring with a calibrated pH meter. Electrical conductivity (EC) was measured using a conductivity meter. Organic matter (%) and nitrogen (%) contents were determined following the Walkley-Black method [52] and the Kjeldahl method [53], respectively. Organic carbon (%), phosphorus (%), and potassium (%) were measured using a total organic carbon analyzer, a spectrophotometer, and a flame photometer, respectively.

#### 2.8.5. Scanning Electron Microscopy (SEM)

Samples (5 × 5 mm) were cut using a dissecting blade, mounted on stub adapters with double-sided carbon tape, and gold-coated for 2 min under high vacuum, following Aiduang et al. [45]. Surface morphology was observed using a scanning electron microscope (TESCAN MIRA, 4th Generation, Tescan Orsay Holding, Brno-Kohoutovice, Czech Republic) operated at 5 kV with TESCAN’s Essence™ software.

#### 2.8.6. Thermogravimetric Analysis (TGA)

Approximately 8 mg of pure mycelium from *P. ciliatus* and *P. subfasciatus* were analysed using a thermogravimetric analysis (TGA) with a thermogravimetric analyzer (TGA/DSC3+, Mettler Toledo, Greifensee, Switzerland), following Cartabia et al. [54]. Dynamic thermogravimetry method was used where samples were heated from 25 °C to 900 °C at a scanning rate of 20 °C min^−1^ under a constant nitrogen purge flow of 25 mL min^−1^.

#### 2.8.7. Fourier Transform Infrared Spectroscopy (FTIR)

FTIR spectroscopy was performed to characterise the chemical composition of the pure mycelium samples using attenuated total reflectance (ATR-FTIR) spectroscopy (Nicolet iS50, Thermo Fisher Scientific Inc., Madison, WI, USA), following Raman et al. [20]. Spectra were recorded in the range of 4000 − 400 cm^−1^ with a resolution of cm^−1^.

#### 2.8.8. Flammability

Flammability testing followed the UL-94 standard for horizontal and vertical burning [55]. Samples (125 × 13 mm) were tested in both orientations. In the horizontal burning test, each sample was held horizontally and exposed to a 20 mm high Bunsen burner flame for 30 s, and the flaming duration and burning length were recorded. In the vertical burning test, the sample was positioned vertically, and the flame was applied to the bottom for 10 s, and flame duration and burning characteristics were recorded. Each test was replicated five times per species.

#### 2.8.9. Soil Burial Degradability

A biodegradability test was performed under soil burial conditions (ISO 846/2000) using fine-grained active soil from forest areas in Chiang Rai Province, Thailand. The soil was sieved to obtain particles smaller than 2 mm [45]. Test samples were wrapped in synthetic netting and buried in soil for 90 days. Five replicates were prepared per treatment. Samples were removed at 15, 30, 45, 60, 75, and 90 days, cleaned, and dried at 60 °C to constant weight. Weight loss was calculated using the formula: WL (%) = (W_i_ − W_t_)/W_i_ × 100, where WL = the percentage of weight loss (%), W_i_ = initial weight (g), W_t_ = weight after the specified time (g).

### 2.9. Product Fabrication

Two separate mycelium-substrate mixtures, consisting of *P. ciliatus* and *P. subfasciatus* with rubber sawdust, were used to develop biomaterial prototypes. Various constructions, household, and decorative items, such as vases, bowls, and blocks, were successfully designed and fabricated.

### 2.10. Viability Maintenance of the Mushroom Strain

The viability of *P. ciliatus* and *P. subfasciatus* strains was maintained through basidiomata formation following the method described by Aiduang et al. [56]. Fresh basidiomata were produced using the same substrate composition as in the test sample preparation. Cultivation bags were incubated in the dark at 25 °C and then transferred to a fruiting chamber at 25 ± 1 °C, 75–80% RH, with illumination. The resulting basidiomata were used for isolation and preparation of a pure culture. Five cultivation bags were used for each species.

### 2.11. Data Collection and Statistical Analysis

Data analysis was performed using the statistical software SPSS v.16.0. Data on mycelial growth rate, physical, mechanical, hydrodynamic, chemical tests, flammability tests, and soil burial biodegradability test were analysed using one-way analysis of variance (ANOVA). An independent samples *t*-test was performed to compare the means of two independent groups. The multiple comparisons of means were deployed when significant differences were found (*p* < 0.05) using Duncan’s Multiple Range Test (DMRT).

## 3. Results

### 3.1. Taxonomy

**Figure 5:**
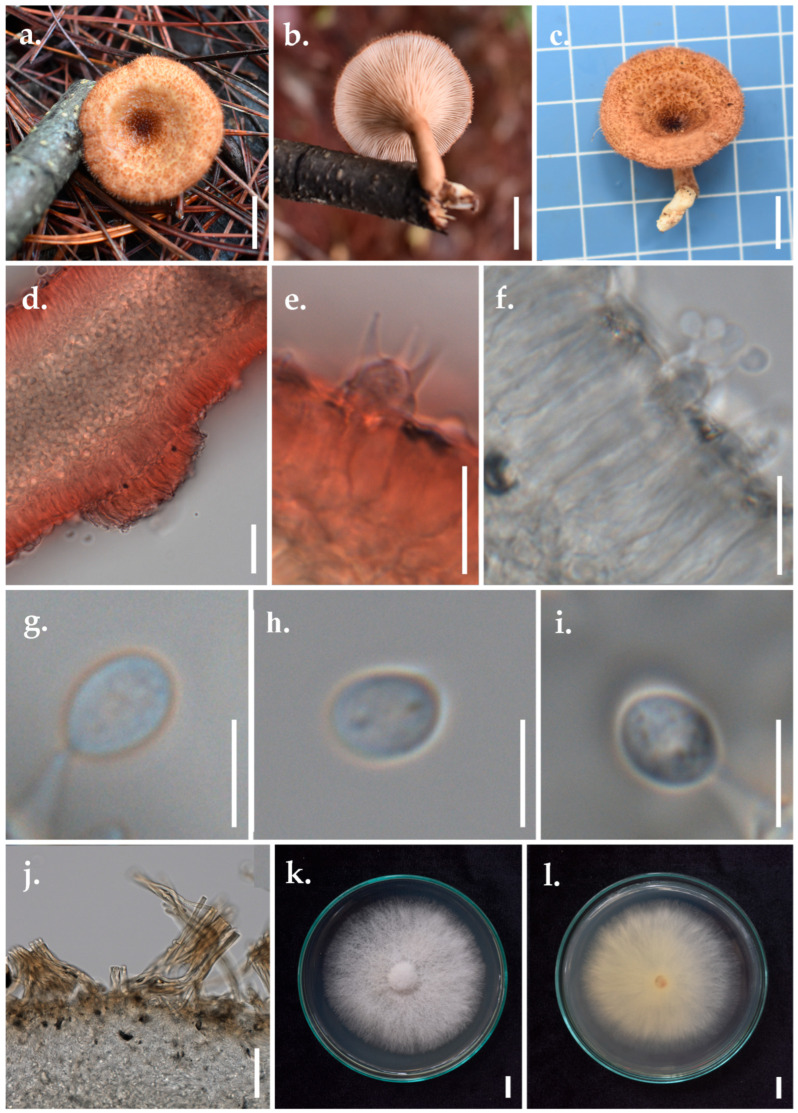
Morphological characteristics of *Panus ciliatus* (MFLUCC 25-0173). (**a**–**c**) Basidiomata. (**d**) Hyphal peg outgrowth in the hymenium. (**e**,**f**) Hymenium showing Basidia. (**g**–**i**) Basidiospores. (**j**) Pileipellis hyphae. (**k**) Culture (top view). (**l**) Culture (bottom view). Scale bar: (**a**–**c**,**k**,**l**) = 1 cm, (**d**) = 20 μm, (**e**,**f**) = 10 μm, (**g**–**i**) = 5 μm, (**j**) = 50 μm.

*Panus ciliatus* (Lév.) T.W. May & A.E. Wood, Figure 5
≡ *Lentinus ciliatus* Lév. *Annls Sci. Nat.*, Bot., sér. 3 2: 175 (1844)

Index Fungorum number: IF 413663; Facesoffungi number: FoF 17547

*Basidiomata* small, stipitate, leathery, solid, and dry, with a densely ciliated margin. *Pileus* 1.7–2.8 cm in diameter, infundibuliform, shallow and concentrically furrowed, membranous. *Pileus surface* tomentose, almost uniformly thick, greyish-orange (6B5–6), brown bristles (7E7), and context negative to KOH. *Margin* entire, incurved, thin, densely ciliated. *Stipe* up to 1.8–5.5 cm long, 3.6–7.2 mm in diameter, cylindrical, tough, almost woody, concolourous with the lamellae, covered with very short hairs. *Lamellae* up to 0.2 mm broad, densely crowded, decurrent, vinaceous (8B4), with 3–4 tiers of lamellulae. *Odour and taste* not distinct.

*Basidiospores* (3.79–)4.27–6.42(–6.73) × (2.87–)3.67–4.95(–5.04) μm (*n* = 30/2), L_m_ = 5.62 μm, W_m_ = 4.29 μm, Q_m_ = 1.31; ellipsoid to cylindric, thin-walled, with few contents. *Basidia* 15–27 × 4–6 μm, clavate, thin-walled, 4-spored, with sterigmata up to 6 μm long; clamps connections present at the base. *Lamella edge* sterile, with abundant cheilocystidia. *Hyphal pegs* present on the lamellar walls, up to 135 μm in diameter, up to 35 μm in length. *Cheilocystidia* 18–24.4 × 5.2–6.4 μm, clavate, hyaline, thin-walled. *Sclerocystidia* abundant on the gill sides, 21.9–32.3 × 3.7–6.7 μm, clavate, hyaline, thin-walled. *Pileipellis* brownish, slightly thick-walled, cylindrical; generative hyphae 4.3–6.1 μm wide, with clamp connections. *Context generative hyphae* 4.4–5 μm wide, hyaline, thin-walled, with clamp connections. *Context skeletal hyphae* 5.1–5.9 μm wide, cylindrical, hyaline, slightly thick-walled.

Habitat: On decaying branches in a mixed deciduous forest.

Material examined: Thailand, Chiang Rai Province, N 20°9′59″, E 99°37′2″, alt. 1200 m, 12 June 2024, S. Khyaju, SK288, MFLUCC 25-0173, MFLU 25-0108.

GenBank Number ITS: PV393836.

Known distribution (based on molecular data): Brazil, China, India, Indonesia, Pakistan, Philippines, Singapore, Sri Lanka, the USA, Vietnam [3], and Thailand (this study).

Note: The morphological characteristics of our specimen are similar to the original description of the holotype from Maluku, Indonesia, which is characterised by its leathery, infundibuliform pileus, concentrically furrowed, tomentose, brown bristles, and sharply defined, densely ciliated margin [57]. However, hyphal pegs were not observed in the descriptions of either the holotype or the Brazilian specimen (SP446150) [3].

**Figure 6:**
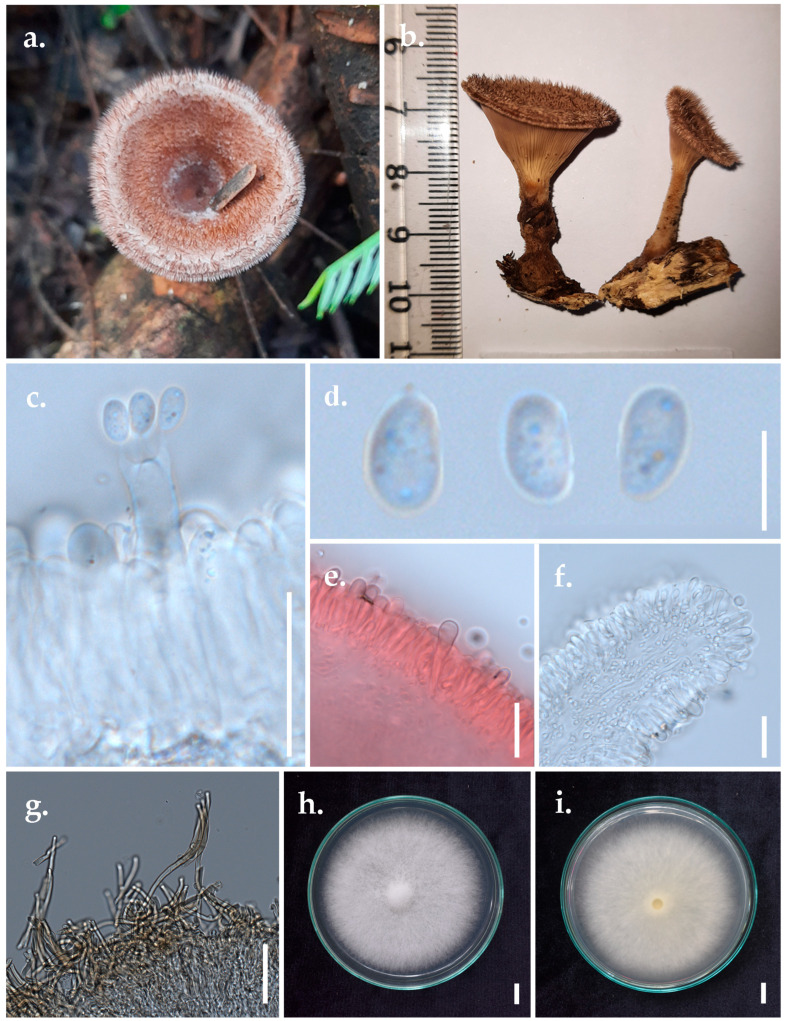
Morphological characteristics of *Panus subfasciatus* (MFLUCC 25-0172). (**a**,**b**) Basidiomata. (**c**) Hymenium showing basidia and basidiospores. (**d**) Basidiospores. (**e**) Hymenium with pleurocystidia. (**f**) Hymenium with Cheilocystidia. (**g**) Pileipellis hyphae. (**h**) Culture (top view). (**i**) Culture (bottom view). Scale bar: (**c**,**e**,**f**) = 20 μm, (**d**) = 5 μm, (**g**) = 50 μm, (**h**,**i**) = 1 cm.

*Panus subfasciatus* Thongbai, Karun., C. Richter & K.D. Hyde, Figure 6

Index Fungorum number: IF 552827; Facesoffungi number: FoF 02915

*Basidiomata* small, stipitate, leathery, solid, and dry. *Pileus* 2–4.5 cm in diameter, thin, coriaceous, and infundibuliform, reddish-brown (8D6–7) to brownish-violet (11D6) when young. *Pileus surface* brown (7D5–6), velvety with numerous hispid squamules forming erect hairs or appearing hispid-strigose, context negative with KOH. Margin entire, incurved, hispid, densely ciliated. *Stipe* 2–5 cm long, 2.7–4.2 mm in diameter, cylindrical, entirely covered with dense, short, soft hairs; dull red (9C4) to greyish-red (9C5) when young, concolourous with the pileus. *Lamellae* up to 0.2 mm broad, with 3–5 tiers of lamellulae, deeply decurrent, brown (7D7), and moderately crowded. *Context* white to light brown (7D5) with purplish shade, composed of a dimitic hyphal system with skeletal hyphae. *Odour and taste* not distinct.

*Basidiospores* (5.55–)6.38(–7.40) × (3.73–)4.00(–4.40) μm (*n* = 30/2) L_m_ = 6.38 μm, W_m_ = 4.00 μm, Q = (1.49–)1.60(–1.68), Q_m_ = 1.60 μm; ellipsoid to elongate, hyaline, thin-walled, with few contents. *Basidia* 20–24 × 3–5 μm, clavate to elongate-clavate, mostly 4-spored, occasionally 2-spored, with sterigmata up to 5 μm long; clamps connections present at the base. *Lamella* edge sterile, with abundant cheilocystidia. *Cheilocystidia* 14–18 × 4–6 μm, clavate, hyaline, thin-walled. *Sclerocystidia* abundant on the gill sides, 23–35 × 5–8 μm. *Pileipellis* interwoven, brownish, slightly thick-walled; generative hyphae 3.0–5.8 μm wide, branched, with clamp connections. *Generative hyphae* included generative hyphae 1.7–3.1 μm wide, hyaline, thin-walled, and branching with clamp connections. *Skeletal hyphae* 2.9–3.7 μm wide, cylindrical, hyaline, and slightly thick-walled.

*Habitat*: On decaying branches in a mixed deciduous forest.

*Material examined*: Thailand, Chiang Rai Province, Muang Chiang Rai District, N 20°3′6″, E 99°53′30″, alt. 422 m, 20 November 2022, S. Khyaju, SK49, MFLUCC 25-0172, MFLU 25-0107.

*GenBank Number* ITS: PV393835.

*Known distribution* (based on molecular data): Cameroon, China, India, Sri Lanka, the USA [3], and Thailand [41].

Note: The morphological characteristics of our specimen are consistent with the original description of the holotype from Thailand, which is characterised by brownish-violet to purplish-brown basidiomata when young, later turning greyish-ruby, leathery, solid, dry, and fully covered with brownish-grey hairs [41]. Micro-morphological features of our specimen also range within the description of the holotype; however, the cheilocystidia and pleurocystidia in our collections are smaller than those reported for the holotype.

### 3.2. Phylogenetic Inferences

**Figure 7:**
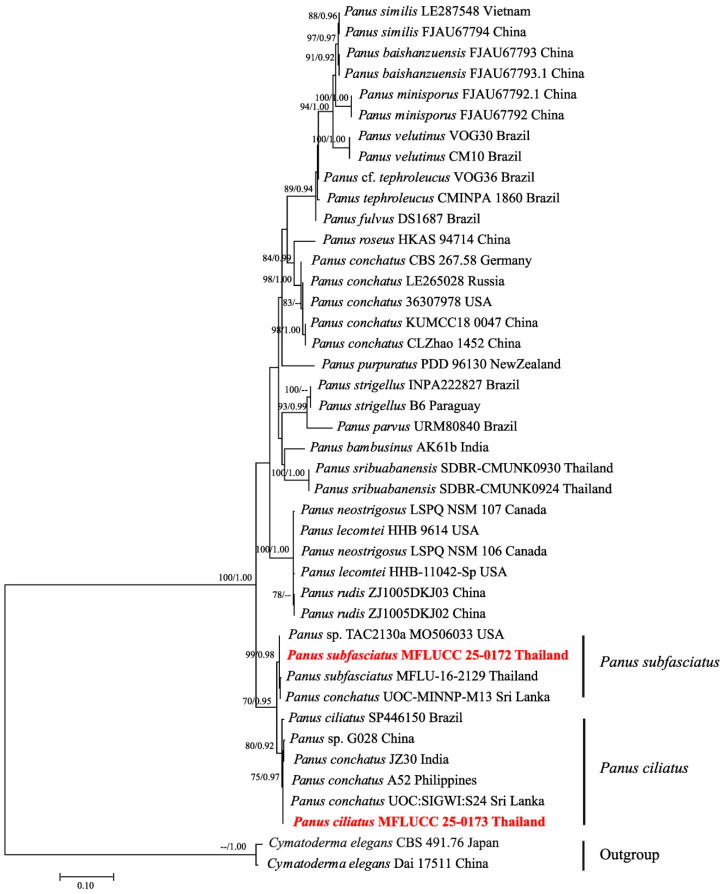
Phylogenetic tree (RAxML) obtained from the DNA sequence data of the ITS dataset. Bootstrap support values (BS) for maximum likelihood (ML, first value) equal to or greater than 70% and Bayesian posterior probabilities from MCMC analyses (BPP, second value) equal to or greater than 0.90 are given above the nodes. The scale bar indicates expected changes per site. The tree is rooted with *Cymatoderma elegans* (Dai 17511) and *C. elegans* (CBS 491.76). Newly generated sequences are indicated in bold red.

Phylogenetic analyses were conducted based on ITS sequences, comprising 42 taxa, including our specimens, additional *Panus* species, and two outgroup taxa: *Cymatoderma elegans* (Dai 17511) and *C. elegans* (CBS 491.76). The final dataset consisted of 581 total characters, including gaps. The best-scoring ML tree is shown in Figure 7, with an optimal log-likelihood value of −2872.843714. The alignment contained 2.54% gaps and undetermined characters. Estimated base frequencies were A = 0.241696, C = 0.219979, G = 0.225115, T = 0.313210; substitution rates AC = 0.668485, AG = 2.660522, AT = 2.057754, CG = 0.741111, CT = 5.494471, and GT = 1.000000. The gamma distribution shape parameter (α) = 0.333169. Phylogenetic analyses placed our sequences of *P. subfasciatus* (ML = 99%, BPP = 0.98) and *P. ciliatus* (ML = 80%, BPP = 0.92) in a well-supported clade. The isolates of both specimens used in this study, collected from Thailand, are presented in bold red.

### 3.3. Effect of Culture Media on Mycelial Growth

**Figure 8:**
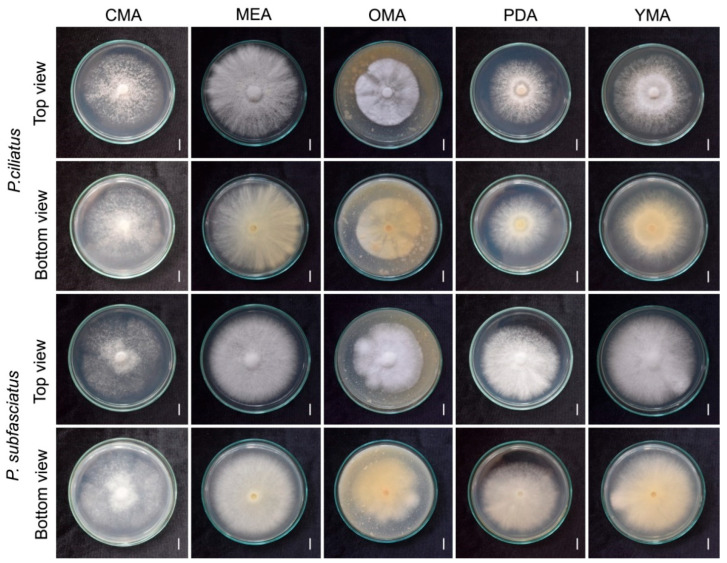
Morphological characteristics of *P. ciliatus* (MFLUCC 25-0173) and *P. subfasciatus* (MFLUCC 25-0172) grown on different agar media at 25 °C for 10 days. Scale bars = 1 cm.

The evaluation of mycelium grown in five different agar media was compared in terms of mycelium morphology, density, and growth rates (mm/day). Among the tested media, MEA exhibited the most favourable growth (Figure 8, Table 2). The mycelium of both species appeared white (6A1) across all media. Colony formation was observed by day 10, with notable differences in density and morphology. For *P. ciliatus*, somewhat abundant growth (4+) was observed on MEA, while CMA and PDA produced a scanty, cottony colony (2+). For *P. subfasciatus*, somewhat abundant growth (4+) occurred on both MEA and YMA, whereas CMA yielded scanty, cottony colonies (2+). Morphologically, *P. ciliates* produced uniformly thick mycelium on MEA and OMA, granular mycelium on CMA, and colonies with thicker centres and thinner edges on PDA and YMA. In *P. subfasciatus*, all media exhibited uniformly thick mycelium except CMA, which showed a granular texture.

Mycelium plug expansion began on day 2. The rapid growth of *P. ciliates* was on MEA (8.24 ± 0.61 mm/day) while *P. subfasciatus* grew most rapidly on both MEA (8.08 ± 0.14 mm/day) and YMA (8.07 ± 0.20 mm/day), covering the entire plates. OMA yielded the slowest growth in both species, *P. ciliatus* with 5.55 ± 0.12 mm/day and *P. subfasciatus* with 6.26 ± 0.14 mm/day.

### 3.4. Mycelium Growth in Lignocellulose Substrate Media

**Figure 9:**
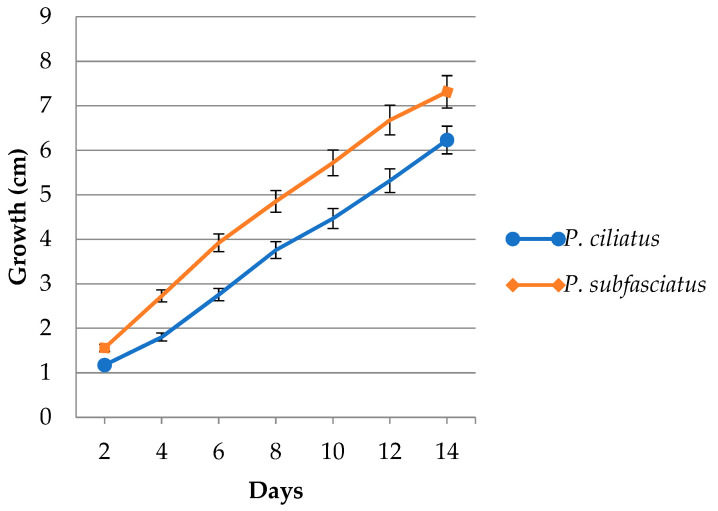
Mycelium growth rate of *P. ciliatus* (MFLUCC 25-0173) and *P. subfasciatus* (MFLUCC 25-0172) on rubber sawdust.

**Figure 10:**
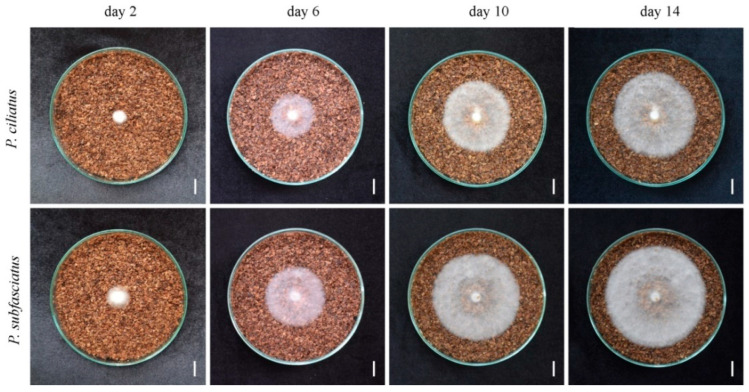
Morphological characteristics of *P. ciliatus* (MFLUCC 25-0173) and *P. subfasciatus* (MFLUCC 25-0172) incubated on rubber sawdust with supplements at 25 °C for 14 days. Scale bars = 1 cm.

The mycelium of both *P. ciliatus* and *P. subfasciatus* was observed to be white and exhibited a regular growth pattern. Colony diameters were measured on alternate days, starting from day 2, for cultures grown on rubber sawdust. The average mycelial growth rates were 4.70 ± 1.35 mm/day for *P. ciliatus* and 6.07 ± 0.89 mm/day for *P. subfasciatus* (Figure 9). On day 14, the mycelial density in the plates of *P. subfasciatus* was abundant (5+) compared to *P. ciliatus*, with somewhat abundant (4+) (Figure 10).

### 3.5. Mycelium-Based Biomaterial Characterisation

#### 3.5.1. Physical Properties

##### Moisture Content and Dry Density

The moisture content of the cube test samples before deactivation was 55 ± 0.51% for *P. ciliatus* and 54.14 ± 0.43% for *P. subfasciatus*. The dry densities of samples from both species were similar, at 272.99 ± 4.44 kg/m^3^ and 273.45 ± 4.87 kg/m^3^, respectively (Table 3).

##### Volumetric Shrinkage

The volumetric shrinkage of cube test samples was 8.54 ± 0.76% for *P. ciliatus* and 8.55 ± 1.35% for *P. subfasciatus* (Table 3).

#### 3.5.2. Mechanical Properties

##### Compression Strength

**Figure 11:**
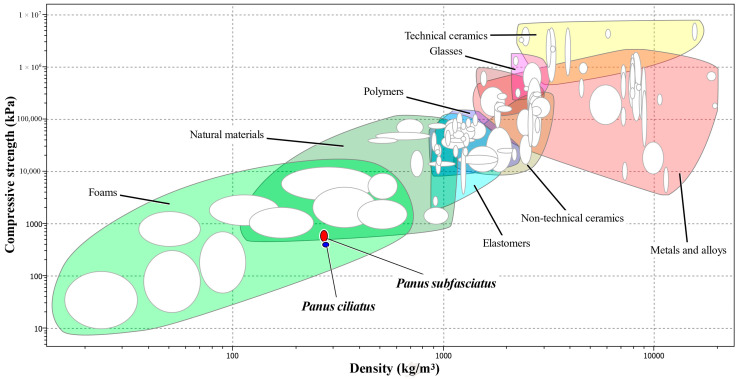
Material property chart (Ashby) comparing MMB samples from *P. ciliatus* (MFLUCC 25-0173) and *P. subfasciatus* (MFLUCC 25-0172) to typical materials families in terms of compressive strength against density. The members of each class are enclosed in envelopes. The image was generated using CES EduPack 2014 v14.3.5.

The compressive strength of MMB samples developed from *P. ciliatus* ranged from 370 to 440 kPa, whereas samples from *P. subfasciatus* ranged from 470 to 730 kPa. On average, MMB from *P. subfasciatus* showed significantly greater compressive strength (604 ± 99 kPa) than MMB from *P. ciliatus* (412 ± 27 kPa) (Figure 11, Table 4).

##### Flexural Strength

The flexural strength of MMB samples developed from *P. ciliatus* ranged from 1110 to 1560 kPa, whereas those from *P. subfasciatus* ranged from 720 to 860 kPa. On average, *P. ciliatus* samples exhibited significantly higher flexural strength (1326 ± 170 kPa) compared to *P. subfasciatus* (800 ± 50 kPa) (Table 4).

##### Tensile Strength

Tensile strength values ranged from 825 to 1106 kPa for *P. ciliatus*, with the average noted to be 989 ± 104 kPa (Table 4).

##### Impact Strength

The impact strength of MMB samples from *P. ciliatus* ranged from 0.09 to 0.16 kJ/m^2^, while *P. subfasciatus* ranged from 0.06 to 0.13 kJ/m^2^. On average, *P. ciliatus* exhibited similar impact strength (0.13 ± 0.03 kJ/m^2^) compared to *P. subfasciatus* (0.09 ± 0.03 kJ/m^2^) (Table 4).

#### 3.5.3. Hydrodynamic Properties

##### Water Absorption

**Figure 12:**
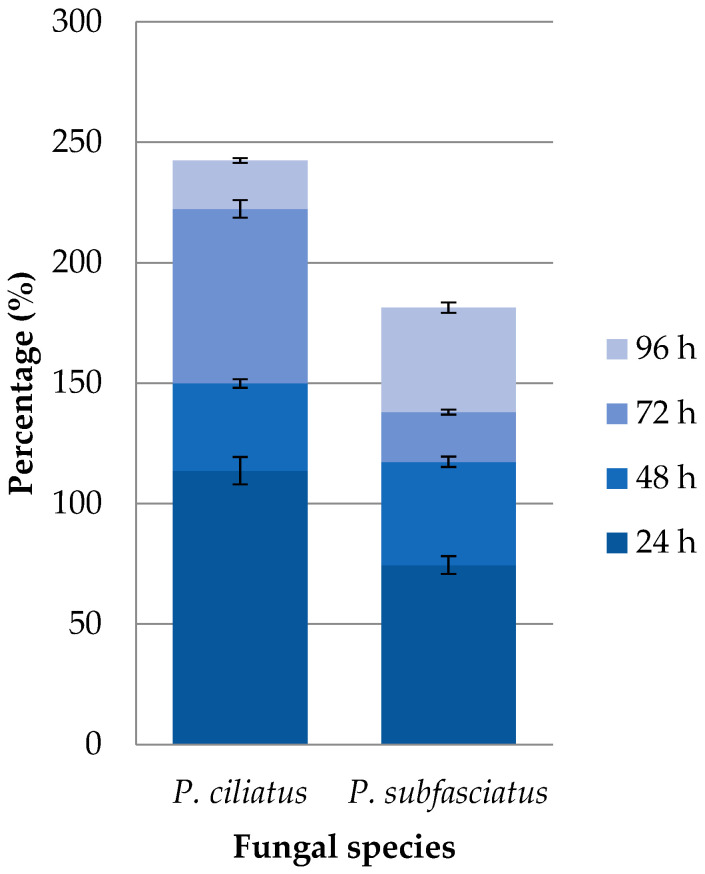
Water absorption of MMB samples from *P. ciliatus* (MFLUCC 25-0173) and *P. subfasciatus* (MFLUCC 25-0172).

The maximum water absorption of cube test samples after 96 h was 242.49% for *P. ciliatus* and 181.41% for *P. subfasciatus* (Figure 12). The water absorption was the highest in the first 24 h, with 113.71% for *P. ciliatus* and 74.52% for *P. subfasciatus*. Overall, *P. ciliatus* exhibited a higher water absorption than *P. subfasciatus*.

##### Water Contact Angle

**Figure 13:**
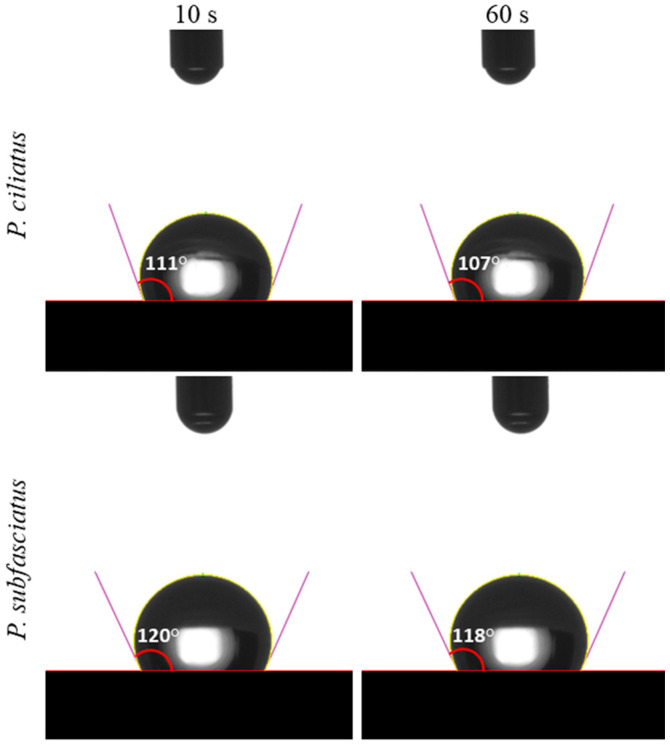
Water contact angle of MMB samples from *P. ciliatus* (MFLUCC 25-0173) and *P. subfasciatus* (MFLUCC 25-0172) at 10 s and 60 s.

Water contact angle (CA) measurements of MMBs developed from *P. ciliatus* and *P. subfasciatus* indicated a hydrophobic behaviour, with CA values exceeding 90° at both 10 and 60 s (Figure 13). At 10 s, CA was 111° for *P. ciliatus* and 120° for *P. subfasciatus*. In 60 s, the CA values slightly decreased to 107° and 118°, respectively.

#### 3.5.4. Chemical Properties

**Figure 14:**
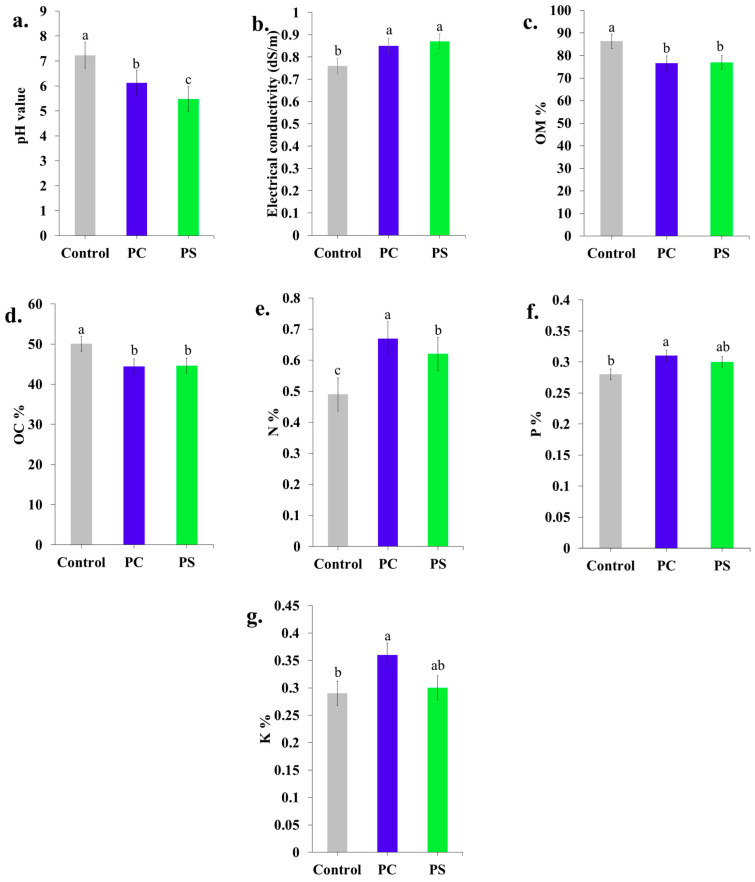
Chemical properties of MMBs produced by *P. ciliatus* (MFLUCC 25-0173) (PC) and *P. subfasciatus* (MFLUCC 25-0172) (PS). (**a**) pH value. (**b**) Electrical conductivity. (**c**) Organic matter content. (**d**) Organic carbon content. (**e**) Nitrogen content. (**f**) Phosphorus content. (**g**) Potassium content. Data are presented as means ± standard error. Bars with the different letter within individual tests are considered significantly different (*p* ˂ 0.05), while bars that share letters ‘ab’ are intermediate and not significantly different from either of ‘a’ or ‘b’, according to Duncan’s multiple range tests.

The chemical properties of the MMBs, including pH, organic matter, organic carbon, nitrogen, phosphorus, and potassium contents, are presented in Figure 14. The pH of the initial substrate (7.23) decreased significantly in MMBs produced by *P. ciliatus* (6.12) and *P. subfasciatus* (5.47), with *P. subfasciatus* exhibiting a greater reduction. The initial EC values of the sawdust substrate before mycelium inoculation were 0.75–0.77 dS/m, which significantly increased in both *P. ciliatus* (0.82–0.88 dS/m) and *P. subfasciatus* (0.81–0.93 dS/m). Organic matter content decreased significantly in MMBs from both *P. ciliatus* (76.63%) and *P. subfasciatus* (76.97%) compared to the initial substrate (86.34%). Similarly, the organic carbon content of the initial substrate (50.08%) decreased significantly in MMBs from both *P. ciliatus* (44.45%) and *P. subfasciatus* (44.64%). While *P. subfasciatus* had a more pronounced pH reduction than *P. ciliatus*, the differences in organic matter and organic carbon content are similar between the two species.

In contrast, nitrogen content increased significantly in MMBs colonised by *P. ciliatus* (0.67%) and *P. subfasciatus* (0.62%) compared to the initial uncolonized substrates (0.49%). Phosphorus content in the initial substrate (0.28%) also increased significantly in MMBs from both *P. ciliatus* (0.31%) and *P. subfasciatus* (0.30%). Similarly, potassium content increased in MMBs from both *P. ciliatus* (0.36%) and *P. subfasciatus* (0.30%) compared to the initial substrate (0.29%). Overall, *P. ciliatus* produced higher levels of nitrogen, phosphorus, and potassium than *P. subfasciatus*.

#### 3.5.5. Scanning Electron Microscopy

**Figure 15:**
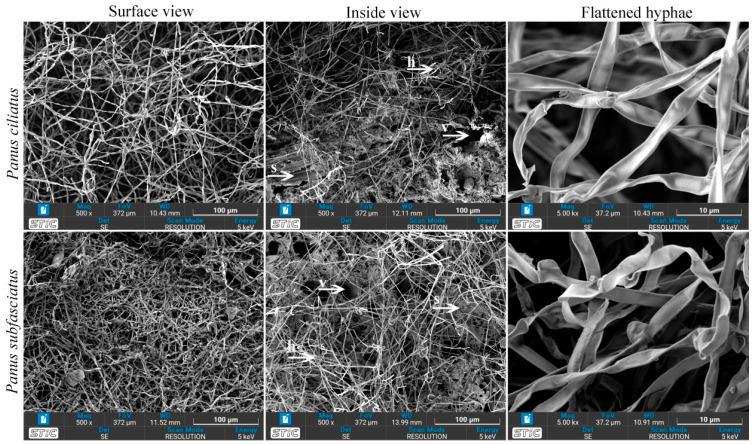
Scanning Electron Microscopy images of MMBs produced by *P. ciliatus* (MFLUCC 25-0173) and *P. subfasciatus* (MFLUCC 25-0172) growth on rubber sawdust. h = hyphae, s = substrate, v = void.

Hyphal structures of *P. ciliatus* and *P. subfasciatus* grown on sawdust substrates were examined using SEM. Both species exhibited similar hyphal morphology (Figure 15). However, visual inspection revealed a higher density of mycelium in *P. subfasciatus* compared to *P. ciliatus*. The top surfaces of the samples were generally covered with loosely interwoven, dense, hairy (fluffy) hyphae. Cross-sectional views showed a mixture of hyphae, voids, and substrate particles. Additionally, the flattened hyphae resulting from hot-air oven dehydrated MMB are also visualised.

#### 3.5.6. Thermogravimetric Analysis (TGA)

**Figure 16:**
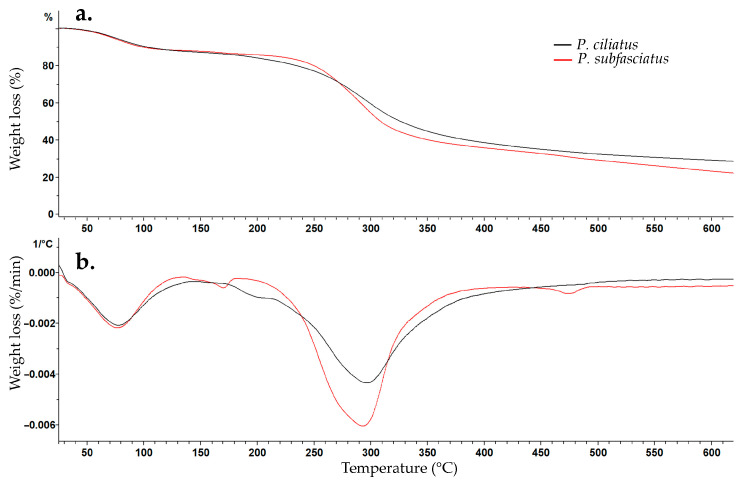
Thermal degradation behaviour of pure mycelium from *P. ciliatus* (MFLUCC 25-0173) and *P. subfasciatus* (MFLUCC 25-0172). (**a**) TGA curves. (**b**) DTGA curves.

The thermal behaviour of pure mycelium from *P. ciliatus* and *P. subfasciatus* is shown by TGA and DTGA curves (Figure 16), exhibiting similar thermal degradation behaviour. The first weight loss occurred at approximately 76 °C, corresponding to moisture evaporation, with losses of 11.65% for *P. ciliatus* and 12.69% for *P. subfasciatus*. The second major degradation phase occurred at 293 °C for *P. ciliatus* (52.89% weight loss) and 298 °C for *P. subfasciatus* (59.94% weight loss). The third event, attributed to the decomposition of residual non-degraded materials and impurities, resulted in 35.47% residual mass for *P. ciliatus* and 27.36% for *P. subfasciatus*.

#### 3.5.7. Fourier Transform Infrared Spectroscopy

**Figure 17:**
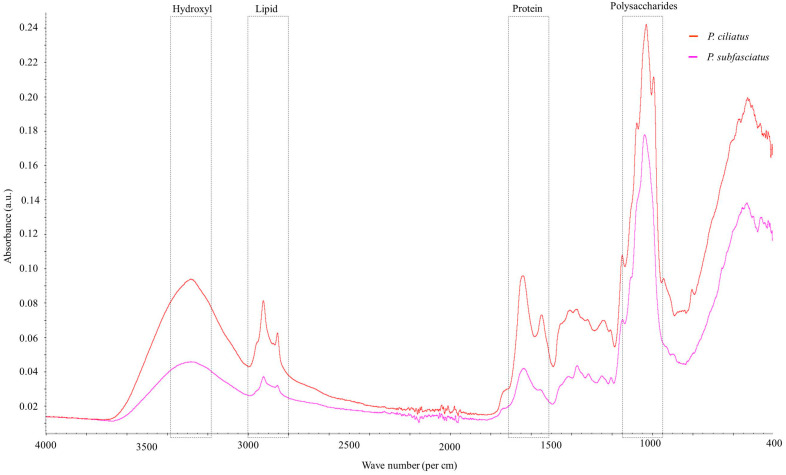
FTIR spectra of pure mycelium from *P. ciliatus* (MFLUCC 25-0173) and *P. subfasciatus* (MFLUCC 25-0172). Major absorption bands corresponding to mycelial cell wall components are labelled.

FTIR spectra of pure mycelium of *P. ciliatus* and *P. subfasciatus* are shown in Figure 17. The analysis revealed characteristic absorption bands corresponding to various chemical constituents. Chemically significant regions in the spectra include the hydroxyl group (3200–3400 cm^−1^), C-H stretching in the fatty acid region (2800–3000 cm^−1^), protein region dominated by C=O (amide I) and N–H (amide II) (1500–1700 cm^−1^), and the polysaccharides region (950–1150 cm^−1^). Details of the peak in the obtained spectra are provided in Table 5.

#### 3.5.8. Flammability Test

**Figure 18:**
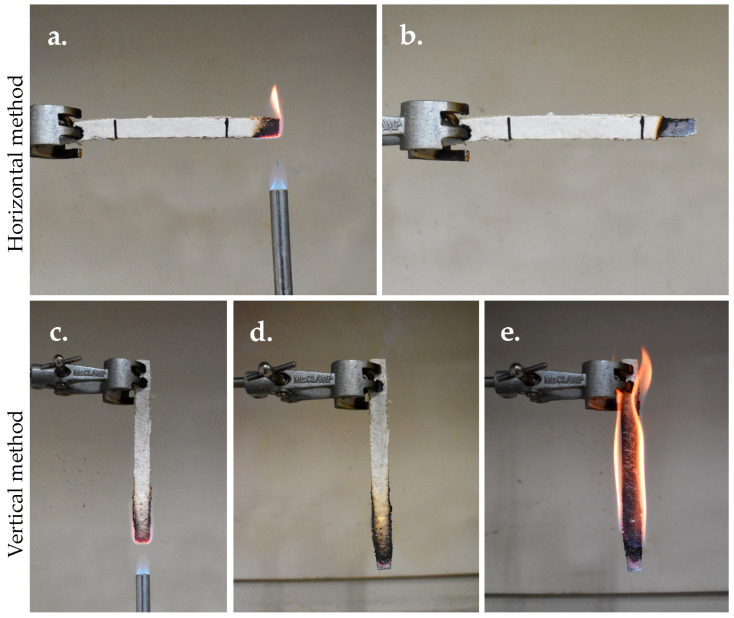
Flammability testing. (**a**) Ignition at the sample tip for 30 s. (**b**) Flame self-extinguished after ignition. (**c**) Ignition at the sample tip for 10 s. (**d**) Flame self-extinguished during the first 10 s of ignition. (**e**) During the second 10 s of ignition, the flame continued to burn and reached the sample holding clamp.

In the horizontal flammability test, following ignition, the flame burned the samples of *P. ciliatus* and *P. subfasciatus* for 21.87 ± 3.87 s and 11.12 ± 1.57 s, respectively (Figure 18). In both cases, the flame did not reach the starting line, and partial burning produced black and grey ash. In the vertical flammability test, after the initial 10 s ignition, the flame burned the samples of *P. ciliatus* and *P. subfasciatus* for 6.62 ± 1.72 s and 5.92 ± 2.57 s, respectively. During the second ignition, the flame extended to consume the entire samples of both species, leaving only ashes.

#### 3.5.9. Soil Burial Degradability

**Figure 19:**
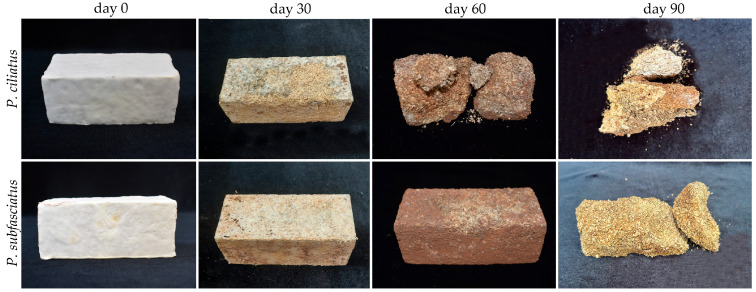
Visual appearance of MMB samples on day 0, 30, 60, and 90 during the soil burial degradation test.

**Figure 20:**
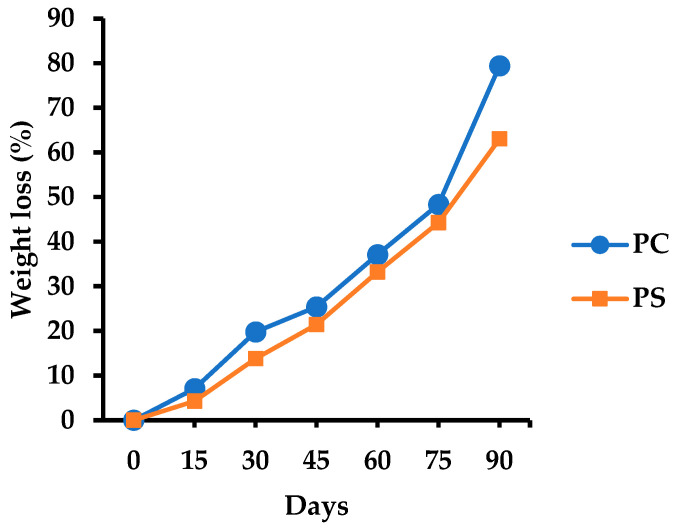
Cumulative percentage weight loss of MMBs samples from *P. ciliatus* (MFLUCC 25-0173) (PC) and *P. subfasciatus* (MFLUCC 25-0172) (PS) during the soil burial degradability test.

The visual appearance of the MMB samples before and after 30, 60, and 90 days of soil burial is shown in Figure 19. All samples gradually disintegrate over time, with the extent of degradation increasing notably by day 90. Initially, the mycelial skin faded, followed by a progressive disintegration into the inner regions. As the burial period increased, the sample sizes decreased. The cumulative percentage weight loss over time is shown in Figure 20. After 90 days of burial, the samples exhibited similar degradation patterns, with an average weight loss of 79.38 ± 9.97% for *P. ciliatus* and 63.02 ± 4.42% for *P. subfasciatus*. Additionally, the colour of the test samples darkened with a prolonged burial.

### 3.6. Developing Prototypes

**Figure 21:**
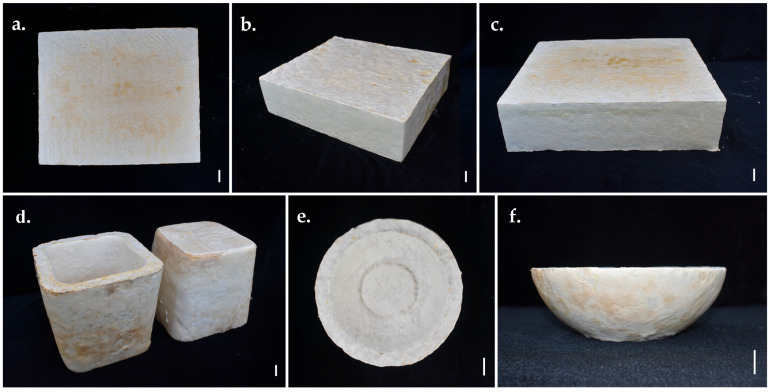
Development of bio-composite prototypes using *P. ciliatus* (MFLUCC 25-0173) grown on rubber sawdust. (**a**–**c**) Block. (**d**) Vase. (**e**,**f**) Circular bowl. Scale bars = 2 cm.

**Figure 22:**
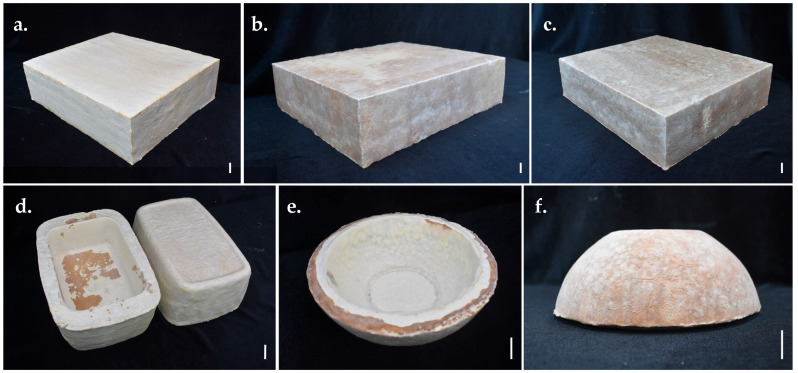
Development of bio-composite prototypes using *P. subfasciatus* (MFLUCC 25-0172) grown on rubber sawdust. (**a**–**c**) Block. (**d**) Rectangular bowls. (**e**,**f**) Circular bowls. Scale bars = 2 cm.

Products from both fungal species exhibited a thick, spongy, white mycelial skin with a brownish shade covering the substrate. Products from *P. ciliatus* included: block (length: 29; breadth: 24.5; thickness: 8 cm), vase (top width: 16.5 cm; bottom width: 13.5 cm; height: 15 cm; thickness: 2 cm), and circular bowl (top diameter: 17.5 cm; bottom diameter: 8 cm; height: 6 cm; thickness: 1.8 cm) (Figure 21). *P. subfasciatus* products included: block (length: 29; breadth: 24.5; thickness: 8.5 cm), rectangular bowl (top length: 23 cm; bottom length: 20.5 cm; top breadth: 15.5 cm; bottom breadth: 13 cm; height: 8.5 cm; thickness: 2 cm), and circular bowl (top diameter: 18 cm; bottom diameter: 8 cm; height: 7 cm; thickness: 1.8 cm) (Figure 22). The mycelium covered all the surface and corners of the products, with no substrate exposed. Additionally, the final products exhibited their unique mycelial skin and showed no fungal contamination.

### 3.7. Viability Maintenance of Mushroom Strains

**Figure 23:**
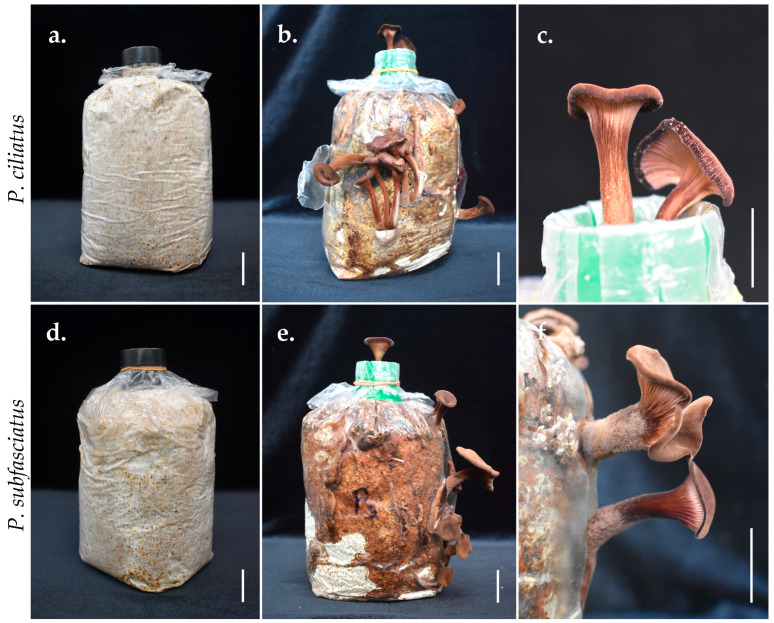
Viability maintenance of mushroom strains. (**a**,**d**) Substrate bag fully colonised by mycelium. (**b**,**e**) Development of basidiomata within the bags. (**c**,**f**) Mature fruiting bodies. Scale bars = 2 cm.

The substrate bags fully colonised by the mycelium of *Panus ciliatus* (MFLUCC 25-0173) and *P. subfasciatus* (MFLUCC 25-0172) that were incubated in the fruiting chamber produced basidiomata (Figure 23). Mature basidiomata were harvested, and fresh tissue was isolated to obtain pure cultures.

## 4. Discussion

Two fungal species collected from forests in Chiang Rai Province, Thailand, were identified as *Panus ciliatus* and *P. subfasciatus* based on a combination of morphological characteristics and ITS phylogenetic analyses. Phylogenetic trees revealed that these two taxa form sister clades with high morphological similarity. Notably, this study reports *P. ciliatus* as a new geographical record in Thailand, thereby expanding the known distribution of the species.

Mycelium growth evaluation on five agar media demonstrated apparent differences in nutritional preferences. *Panus ciliatus* exhibited the fastest growth on MEA with abundant mycelial mat formation, while *P. subfasciatus* grew equally well on MEA and YMA. Although previous studies on *Panus* species are limited, our results support MEA as a favourable substrate, likely due to its high carbon and protein content. On rubber sawdust, both *Panus* species colonised successfully; however, *P. subfasciatus* exhibited a faster growth rate, indicating potential species-specific adaptations to lignocellulosic substrates.

The MMBs produced from these species exhibited physical and chemical properties comparable to those reported in other fungal composites [64]. Their relatively low densities (272.99 and 273.45 kg/m^3^) fall within the range of previously studied MMBs and are lighter than conventional wood products while still denser than synthetic foams.

Shrinkage values (8.54–8.55%) are within the typical range for fungal composites and lower than many wood-based materials, suggesting good dimensional stability. However, compressive strength values were at the lower end of published ranges (0.03–4.44 MPa), likely reflecting the absence of hot or cold pressing during fabrication. Previous work has shown that pressing can significantly enhance mechanical properties, allow additional densification, and improve performance in structural applications [65,66]. The strength of the MMBs can also be attributed to their composition, mycelium growth, density, and porosity. Nevertheless, flexural, tensile, and impact strengths of the *Panus*-based MMBs were comparable to those of polystyrene foams and natural materials, supporting their potential as sustainable alternatives for non-load-bearing uses.

Analysis of the Ashby chart reveals that the mechanical properties of MMB reside in the intersection region between natural materials and foams, thereby implying that the MMB material investigated in this study exhibits mechanical properties appropriate for use as a foam substitute. The observed differences in the mechanical properties of MMB from *P. ciliatus* and *P. subfasciatus* are likely attributable to variations in their microstructural and biochemical composition. The superior compressive strength of MMB from *P. subfasciatus* is consistent with its higher mycelial density, as confirmed by SEM analysis. The presence of chitin fibrils in the fungal cell walls is believed to be a primary contributor to this strength by resisting crack propagation under compression [67,68]. Conversely, the enhanced flexural and impact strength of MMB from *P. ciliatus* may be influenced by its protein and lipid content. The higher lipid content in *P. ciliatus*, as indicated by FTIR (Figure 17), likely results in a less rigid mycelial matrix compared to *P. subfasciatus*. This increased flexibility, despite high polysaccharide signals, directly contributes to *P. ciliatus*’s lower compressive strength. Conversely, *P. subfasciatus*’s superior compressive strength appears to be driven by its higher mycelial density, providing a more robust structure. These components can act as plasticizers, imparting greater flexibility and toughness to the material [58]. To confirm the plasticizer effect, further study on protein and lipid content is required.

Hydrodynamic properties also suggest potential for real-world use. The water absorption capacity of *P. ciliatus* and *P. subfasciatus* is within the broad range reported for other fungal composites (24.45–560%) but was higher than that of synthetic polymers [47,49,69]. The lower water absorption in these samples likely reflects the use of fine sawdust particles (≤2 mm), which increases density and reduces porosity [48,70]. In contrast, MMBs absorb more than synthetic polymers (0.01–9%) [65,71]. They demonstrated acceptable hydrophobicity (contact angles > 100°) [58]. The proteins, such as mannoproteins and hydrophobins, present on the outermost layer of the fungal cell wall, are responsible for the hydrophobic nature of MMBs [72,73]. This hydrophobic nature is an important property that benefits product development.

Chemical analyses indicated that MMBs produced from both species contained elevated nitrogen, phosphorus, and potassium relative to the initial substrates. The pH values (4.67–6.12) are consistent with prior studies [19,49,51], with reductions attributed to the enzymatic activity of the fungal mycelium [53]. On the other hand, the electrical conductivity (EC) increased in colonised substrates, likely due to the enzymatic release of minerals [45,74,75]. Macronutrient analysis revealed elevated nitrogen, phosphorus, and potassium (NPK) contents in MMBs compared to the initial substrate, particularly in *P. ciliatus* (N:P:K = 0.67:0.31:0.36), which is higher than *P. subfasciatus* (N:P:K = 0.62:0.30:0.30). The nitrogen content obtained in this study (0.5–1.6%) is consistent with previous studies [53,69,76]. The increased level of NPK is likely due to the enzymatic digestion and fermentation of substrates by mycelium [51]. The significant reduction in OM and OC content observed, as in earlier studies, indicates the inherent capacity of mycelium to modify lignocellulosic substrates derived from agricultural and forestry sectors [49,53]. Following utilisation, MMBs can be easily composted and used as fertilisers in the agricultural sector, thereby reducing production costs and pollution.

Morphological observations via SEM revealed dense, interwoven hyphae binding sawdust particles, functioning as a natural adhesive. Frequent branching of hypha, that results in a dense mycelium mats, indicates its efficiency in colonisation and penetration of the substrate. The cross-linked hyphal strands might contribute in structural integrity and strength of the final material. Voids within the structure explain water absorption through capillary action, while flattened hyphae indicate shrinkage during drying. Such microstructural features directly influence density, porosity, and strength.

Regarding thermal analyses, TGA and DTGA results showed thermal behaviour for both the *Panus* species. The first phase of weight loss (~12%) that occurred near 76 °C was due to the removal of physically and chemically bonded moisture and volatile compounds from the samples [77,78,79]. The major second-phase weight loss (52.89–59.94%) occurred between 293 and 298 °C, corresponding to the decomposition of cellulose, hemicellulose, and lignin, into volatile forms [79]. The results in this study are similar to those in previous studies (220 °C and 410 °C) [21,78]. The third phase is associated with non-degraded impurities, represented in the form of char [78,79]. In this study, char residues were higher in *P. ciliatus* (35.47%) than in *P. subfasciatus* (27.36%), suggesting greater thermal stability and fire resistance. Chitin and chitosan in fungal cell walls may contribute to the thermal behaviour by changing the composition of lignocellulosic substrates following colonization [64,79,80,81]. FTIR spectroscopy revealed the hydroxyl, lipid, protein, and polysaccharide functional groups, with *P. ciliatus* showing stronger signals for certain bonds than *P. subfasciatus*.

Fire resistance is a key requirement in transportation, building, insulation, or packaging, and MMBs show potential in these areas. Flammability tests demonstrated intrinsic fire-retardant properties, with flames self-extinguishing during both horizontal and vertical trials. This behaviour is attributed to char formation and the presence of dense mycelial mats, which limit oxygen penetration [82,83]. During pyrolysis, phosphorus-containing compounds effectively form acids that promote dehydration and char formation [79]. Additionally, the hyphal surface proteins, hydrophobins, also promote char formation [84]. These findings suggest MMBs are safer alternatives to highly flammable polymer foams due to the inherent characteristics of mycelium in the former, indicating their suitability in a wide range of applications, including transportation, packaging, indoor designs, and insulation materials.

The MMBs are considered environmentally friendly and economically viable, relying on the sustainable sourcing of resources [21]. Biodegradability test of samples produced from both *Panus* species demonstrated that the weight loss exceeded 60%, thereby confirming their biodegradable nature. The disintegration of MMBs in soil is primarily driven by the progressive breakdown of mycelium [85]. The observed weight loss can be attributed to multiple processes, including microbial activity, biochemical reactions, and compound leaching [76,85]. MMBs undergo rapid decomposition in soil, adhering to the principles of the circular economy. Additionally, it serves as an alternative to petroleum-based materials (plastics and foams) in packaging, building, indoor uses, and insulation, which are non-biodegradable, environmentally polluting, and pose threats to people and wildlife.

Species of the genus *Panus* have not yet been reported in MMB development. This is the first successful application of mycelium from *Panus* species in combination with rubber sawdust in the design and development of construction and household items, such as blocks, vases, and bowls. Therefore, it highlights the practical utility of these fungi in the production of biomaterials. These laboratory-scale findings indicate the potential of *Panus* species for large-scale MMB production, provided suitable manufacturing resources, financial resources, and experts are available. Additionally, the genetic modification in fungal species, such as the introduction of the glyceraldehyde-3-phosphate dehydrogenase (GPD) promoter into *Ganoderma* species, resulted in 135–165% higher ꞵ-glucan content, thereby enhancing compression strength [86,87]. Similarly, for commercial production and applications, like other fungal genera (*Ganoderma*, *Pleurotus*, *Trametes*), further investigation to strengthen the mechanical properties through reinforcement and pressing could be beneficial.

Maintaining viable fungal cultures is essential for the successful MMB production. Repeated subculturing often leads to strain degeneration [88,89,90]; therefore, in this study, basidiomata formation was stimulated to allow re-isolation of cultures. This approach supports long-term stability and genetic fidelity of production strains and is recommended for integration into large-scale manufacturing processes.

Overall, this study provides the first integrated report on *P. ciliatus* and *P. subfasciatus* as promising fungal resources for MMB production. Their composites display several favourable properties, including low density, dimensional stability, hydrophobicity, thermal stability, fire resistance, and biodegradability. Mechanical strength remains a limitation. Targeted improvements, including pressing, substrate reinforcement, changing cultivation methods, and genetic recombination techniques, could enable these materials to become competitive, sustainable alternatives to petroleum-based foams and plastics at industrial scales.

## 5. Challenges and Practical Recommendations

Although the prototypic MMBs, using *Panus ciliatus* and *P. subfasciatus* and sawdust, have been successfully developed, industrial-level production remains a challenge. Maintaining hygienic environments and sterilizing equipment requires a carefully, timely, and orderly managed industrial setup. Well-trained technicians play a crucial role in operating the production sector efficiently and effectively, indicating the necessity of experts in this industry.

Before deciding on investment in MMBs production, a preliminary economic cost–benefit analysis is important. Regarding lignocellulosic substrate, agricultural and forest residues serve as an accessible, affordable, and appropriate source. Additionally, fungal strains can be sourced either from wild or commercial sources. However, laboratory settings require a significant financial investment for equipment and electricity to manage the environment, including temperature, relative humidity, and hygiene.

Understanding the demand and supply of MMBs in the market, like other products, is a key factor in determining the price. MMBs are considered cost-competitive with any other commercial foam [17]. The price rationality, accessibility, and availability of materials directly affect the introduction, expansion, and penetration of the MMB industry in new locations. However, the growing consciousness of sustainability has triggered demand from national and international companies [17]. Being a novel material, its market value obviously faces both risks and uncertainty, demanding a thorough preliminary market study.

Many published research papers have reported the characteristics of MMBs based on final materials without proper identification of the fungus at the species level. This misleading research could be corrected through the scientific identification of fungal species, based on morphological and molecular phylogenetic analyses that provide a strong foundation for understanding material properties. This approach could support knowing the varying properties of different fungal species, both in pure mycelium materials and composite materials.

Another undeniable challenge in the MMB industry is the improvement and standardization of production methodology. Mycelium growth duration, mould dimensions, deactivation conditions, and testing methods greatly impact the material properties, uses, and durability. This limitation could be addressed with an increase in in-depth research and development and setting protocols in alignment with internationally accepted standards, such as ASTM and the International Organization for Standardization (ISO). Additionally, the enhancement of the mechanical strength of MMBs is possible only with intensive, interdisciplinary, and collaborative research efforts.

## 6. Conclusions

MMB development utilises lignocellulosic byproducts and mycelium to produce highly beneficial and value-added materials. In this study, *Panus ciliatus* and *P. subfasciatus* were identified, the former species being a new geographical record in Thailand (ML = 80%, BPP = 0.92). MMB developed using these species exhibited suitable physical and mechanical properties, supporting previous studies and matching synthetic foams. However, the low degree of mechanical properties exists as a challenge for materials. The lower degree of shrinkage (around 8%) of MMB material supports construction and packaging purposes. The water contact angle above 100 degrees with the samples reflects the hydrophobicity of the mycelium of these species. However, the higher level of water absorption by MMB suggests their suitability in dry conditions for packaging and indoor applications. Thermal behaviour analysis and flammability showed greater thermal stability due to formation of char and resistance for heat transmission. The higher percentage (above 60%) of weight loss in 90 days proved MMB to be a biodegradable and eco-friendly material. Both chemical changes and biodegradability showed that composting MMB contributes to ecological balance through both nutrient cycling and soil enrichment. Additionally, viability maintenance technique for mycelium culture was successfully conducted, providing a solution for culture degeneration issues. The prototypic MMB provides insights for the commercial-scale production potential of these two mushrooms, including construction, packaging, household uses, and insulation products.

## Figures and Tables

**Table 2:** Effect of different agar media on the mycelial growth rate and mycelial density of *P. ciliatus* (MFLUCC 25-0173) and *P. subfasciatus* (MFLUCC 25-0172) was studied by incubating them at 25 °C for 10 days.

Culture Agar Media	Mycelium Growth Rate (mm Day^−1^)		Mycelium Density	
	*P. ciliatus*	*P. subfasciatus*	*P. ciliatus*	*P. subfasciatus*
CMA	7.07 ± 0.14 ^b^	7.77 ± 0.13 ^b^	2+	2+
MEA	8.24 ± 0.61 ^a^	8.08 ± 0.14 ^a^	4+	4+
OMA	5.55 ± 0.12 ^d^	6.26 ± 0.14 ^c^	3+	3+
PDA	6.45 ± 0.06 ^c^	7.70 ± 0.34 ^b^	2+	3+
YMA	7.15 ± 0.28 ^b^	8.07 ± 0.20 ^a^	3+	4+

Note: Within a column, values followed by the same letter are not significantly different (*p* < 0.05) by Duncan’s Multiple Range Test.

**Table 3:** Moisture content, dry density, and volumetric shrinkage of MMB samples obtained in this study.

Parameter	*P. ciliates* (MFLUCC 25-0173)		*P. subfasciatus* (MFLUCC 25-0172)	
	95% Confidence Interval	Average	95% Confidence Interval	Average
Moisture content (%)	54.38, 55.63	55 ± 0.51	53.61, 54.67	54.14 ± 0.43
Dry density (kg/m^3^)	267.48, 278.51	272.99 ± 4.44	267.41, 279.50	273.45 ± 4.87
Volumetric shrinkage (%)	7.60, 9.49	8.54 ± 0.76	6.88, 10.22	8.55 ± 1.35

Note: Values represent mean ± standard deviation. Within a row, means are not significantly different (*p* ˃ 0.05) by the independent samples *t*-test.

**Table 4:** Compressive, flexural, tensile, and impact strengths of MMB samples obtained in this study.

Parameter	*P. ciliatus* (MFLUCC 25-0173)		*P. subfasciatus* (MFLUCC 25-0172)	
	95% Confidence Interval	Average	95% Confidence Interval	Average
Compressive strength (kPa)	377.55, 446.46	412 ± 27.75 ^a^	479.96, 728.04	604 ± 99.90 ^b^
Flexural strength (kPa)	1113.90, 1538.10	1326 ± 170.82 ^a^	737.30, 862.70	800 ± 50.50 ^b^
Tensile strength (kPa)	859.34, 1117.95	988.65 ± 104.14	–	–
Impact strength (kJ/m^2^)	0.08, 0.17	0.13 ± 0.04 ^a^	0.06, 0.12	0.09 ± 0.03 ^a^

Note: Values represent mean ± standard deviation. Within a row, values followed by the same letter are not significantly different (*p* < 0.05) by the independent samples *t*-test. “–” not available due to contamination in samples.

**Table 5:** Characteristic absorption bands observed in the FTIR spectra (4000−400 cm^−1^) of *P. ciliatus* (MFLUCC 25-0173) and *P. subfasciatus* (MFLUCC 25-0172).

Wave Number Range (cm^−1^)	Spectra	Peak (cm^−1^)		References
		*P. ciliatus*	*P. subfasciatus*	
3600–3000	O−H stretching hydrogen bonds	3281	3282	[47,58]
3000–2840	C–H stretching in methyl and methylene groups	2922	2921	[47,59,60]
2940–2840	CH_2_ asymmetric stretching	2853	−	[47]
2900–2850	CH_3_ and CH_2_ stretching	2853	–	[61]
1658–1625	Amide I; chitin	1640	1633	[61]
1560–1520	C=C stretching of aromatic ring (syringyl) in lignin	1547	−	[47]
1460–1400	CH_2_ and CH_3_ deformation in the lignin and hemicellulose; CH_2_ in-plane bending vibrations in the cellulose and lignin	1405	−	[62]
1370–1365	Aliphatic C–H stretch in CH_3_	1370	1370	[60]
1375–1315	O–H bending polysaccharide; Amide III	−	1313	[61]
1250–1025	C–O bond; β (1 → 3) glucan; cell wall polysaccharide	1241, 1075	1248	[61]
1205–1200	OH plane deformation in cellulose; C−C stretching, C–O stretching, C−H deformation	−	1202	[58,60]
1162–1125	C–O–C asymmetric valence vibration	1146	1145	[59,60]
1060–1015	C–O valence vibration from C_3_–O_3_H	1031	1039	[60]
1047–1004	C_alky_–O ether vibrations	1028	1036	[60]
1185–900	Polysaccharides	992	−	[63]
950–700	Glycosidic bond β–(1 → 4) arabinogalactans, galactomannans, and cellulose	933, 943	−	[62]

Note: “–” not available.

## Data Availability

The original sequencing data presented in the study are openly available in GenBank under accession numbers PV393836 and PV393835. The original contributions presented in this study are included in the article. Further inquiries can be directed to the corresponding author.
